# Nutrigenomics-Associated Impacts of Nutrients on Genes and Enzymes With Special Consideration of Aromatase

**DOI:** 10.3389/fnut.2020.00037

**Published:** 2020-04-09

**Authors:** Helena Jenzer, Leila Sadeghi-Reeves

**Affiliations:** ^1^Department of Health Professions, aR&D in Nutrition and Dietetics, Bern University of Applied Sciences BFH, Bern, Switzerland; ^2^Internistic Service, Hospital Pharmacy, Hospital of Psychiatry, University of Zurich, Zurich, Switzerland

**Keywords:** nutrients, nutrigenomics, aromatase, CYP19A1 isoenzyme, food-drug interactions, healthy aging, personalized nutritional medicine, flavonoids

## Abstract

Interactions are occurring in the course of liberation, absorption, distribution, metabolism, and excretion of active ingredients, or at the target receptors. They are causing therapy failures and undesirable events. Forty-seven of fifty-seven human hepatic isoenzymes are specific and relevant in hormone and vitamin metabolism and biosynthesis. Aromatase (syn. CYP19A1) is one of the specific CYP450 isoenzymes so far not elucidated in detail. As aromatase-inhibiting phytochemicals are currently recommended for breast cancer prevention and as add-on accompanying aromatase-inhibitor pharmacotherapy, it was the aim of this literature review to assess whether a common interpretation on genetic and -omics basis could be found. Articles retrieved showed that traditional antioxidation diet is one of the most approved explanations of inhibition of aromatase by phytonutrients of flavonoid derivatives. Flavonoids compete for the oxygen provided by the heme moiety of aromatase in the course of aromatase-catalyzed conversion of steroid precursors to estrogens. Flavonoids are therefore promoted for breast cancer prevention. A further explanation of flavonoids' mechanism of action proposed was related to enzymatic histone deacetylation. By keeping DNA-structure wide through a high acetylation degree, acetylated histones favor transcription and replication. This mechanism corresponds to a procedure of switching genes on. Inhibiting acetylation and therefore switching genes off might be an important regulation of repressing cancer genes. Aromatase expression depends on the genotype and phenotype of a person. Aromatase itself depends on the expression of the heme moiety encoded in the genotype. Biosynthesis of porphyrins in turn depends on the substrates succinate and glycine, as well as on a series of further enzymes, with ALA synthetase as the rate-limiting step. The effect of the heme moiety as prosthetic group of aromatase further depends on the absorption of iron as a function of pH and redox state. To assess the function of aromatase precisely, multiple underlying biochemical pathways need to be evaluated. As a conclusion, the genetic regulation of metabolism is a complex procedure affecting multiple pathways. To understand a metabolic step, multiple underlying individually performing reactions need to be considered if personalized (nutritional) medicine should bring an advantage for a patient. Nutrition sciences need to consider the genome of an individual to truly find answers to nutrition-derived non-communicable diseases. With current GWAS (genome-wide association study) approaches, inherited errors of metabolism are identified and ideally treated effectively. It is much more difficult to get a precise genetic profile for non-communicable diseases stemming from multifactorial causes. Polygenic risks evaluation is feasible but diagnostic tools are not yet available in a desired extent. Neither flavonoid researchers nor providers of genetic testing kits are going into the details needed for a truly personalized nutritional medicine. The next step with profiling the exome and then the whole genome is on the threshold of becoming routine diagnosis and of bringing the desired details.

## Introduction

Healthy aging, individual lifestyle, and personalized medicine are an attempt to square the circle. Instead of a progredient decline of an aging person's health, disability-free survival is expected to persist until the person's sudden death. It is not well-understood how disability-free survival can be achieved, whether to just live and accept one's fate or to order a genetic testing and apply suitable preventative measures.

There are many examples of senior athletes still able to perform at highest levels, sometimes to break rules such as “they will never come back.” Playing tennis in the top 10 even at an age of almost 40 years, playing in ice-hockey top leagues until aged more than 40 years, have been proven to be feasible. Obviously, adaption is one of the keys to remain competitive (1). It is however more difficult for ordinary people to maintain health and performance at later phases of life.

To prevent a progredient decrease in the quality of life, various recommendations are brought to the market often for commercial purposes and seldom for truly providing more quality of life nor evident added value (2, 3). Nutritional behavior might contribute to deteriorate chronic non-communicable diseases. Yet, simple associations with disease incidences are hardly ever convincingly evident. Overrepresented old publications are often taken as standard although biases and confounding factors (4).

Healthy aging might depend on a well-tuned adaption of an eating behavior to an individual's metabolism. Apart from diet, physical activity is promoted to maintain a person's and the population's health. However, one-fits-all strategies such as the “eat the rainbow” mantra or the Swiss Food Pyramid do not consider personalized nutritional medicine (5). Recommended daily allowances, e.g., DACH recommendations (6), are based on epidemiological data arising from the need of defined small healthy population groups. These general needs are corrected by two standard deviations and additional amounts to compensate losses from bad accessibility to nutrients from foodstuff or from processing. In addition, special physiological conditions such as pregnancy justify an extra supplement. Recommended daily allowances are therefore unprecise from a nutritional medicine point of view as they do not consider adequately individual needs.

Nutrition research is under pressure due to poor methodologies. Solutions will not come from more and more papers based on qualitative research or from small randomized trials (7). Even the Predimed study which has shown a risk reduction by intake of olive oil and nuts cannot be transferred simply from the Spanish study population to other ethnicities with different nutritional behaviors (8). Therefore, methods have to be expanded or changed. Suitable approaches such as the use of OMICS technologies show promising results in finding reasons why individuals response differently to the same foodstuff or consumption of plant food^1^.

Consuming a number of diets without reflection is not a suitable strategy for healthy aging. Once food and medicines are consumed, interactions are occurring in the course of liberation, absorption, distribution, metabolism, and excretion of active ingredients, or at the target receptors. They are causing therapy failures and undesirable events. As only some 10 of 57 human hepatic non-specific CYP450 isoenzymes are considered important in drug—drug interactions, the expansion of research to specific CYP450 isoenzymes relevant for food-drug interactions, such as aromatase (syn. CYP19A1, EC 1.14.14.1), is a neglected issue in nutrition research. As phytochemicals are currently recommended for breast cancer prevention and as add-on accompanying aromatase-inhibitor pharmacotherapy, a fresh look at the favorite mechanisms is needed.

The aim of this review is:

- To shed light on the link between non-communicable diseases and underlying nutritional behaviors as well as on the inheritability of metabolic performance and their impact on these diseases
- To assess whether explanations beyond pure chemical mechanisms of action are described in the literature and whether a common interpretation based on genetic and omics technologies is found.

## Methodology

A semi-systematic online literature search was performed on the platforms Medline®, Sciencedirect®, Embase®, and Scholar Google® using mesh terms and keywords for several topics of interest. The review strategies including the search and selection of the articles are based on PRISMA guidelines for Systematic Reviews and Meta-Analyses (9). The studies were related to the influence of nutrients on cytochrome P450 and how diet can affect metabolic biochemical pathways. Information was interpreted immediately. New keywords were defined for a step-by-step incremental progress and reentered into the search engines for a second and third run on each of the search chapters.

Firstly, basic search was performed on pharmacokinetics and nutrients, substrates, inhibitors, inducers of human CYP450 isoenzymes, CYP19A1, aromatase, gender, pharmacodynamics and targets of bioactive, food-drug interactions, nutrigenomics, aromatase, and flavonoids. A literature research yielded 2954 bibliographic records in the first run with “[(breast cancer) AND (cytochrome P450)]” keywords and 5307 with “(cytochrome P450) AND nutrients.” Duplicates were identified and removed from the pool of bibliographic records. Next, an accurate screening of abstracts and titles was performed to determine the most relevant articles. Afterwards, full articles were reviewed using the following inclusion criteria: (1) studies which provided information about the relationship between nutrition and breast cancer; (2) studies which explained the metabolic pathways of cytochrome P450 enzymes; (3) studies which explained how some nutrients interact with substrates metabolized by cytochrome P450 enzymes; (4) studies which provided clinical information about the pathogenesis of breast cancer; (5) studies which provided information about any cytochrome P450 enzyme. In conclusion, 52 studies were first retained, mostly recent studies but also some earlier studies presenting analyses that were not found in the most recent studies.

Secondly, the mechanistic pattern was semi-systematically expanded to mechanistical explorations on specific enzymology information about aromatase, breast cancer, breast cancer associated to nutrition, cytochrome P450 associated to breast cancer, nutrition and cancer, natural aromatase inhibitors, risk factors for breast cancer, complementary therapies, and breast cancer.

Thirdly, non-systematic specific search terms on nutrigenomics and biochemical ascending and descending pathways in the aromatase metabolism were searched.

Integration was approached in deductive reflection rounds. Substantial bricks were assembled to a comprehensive nutrition-relevant pattern of personalized nutritional medicine.

## Results and Interpretation

### Pharmaco- and Nutrient-Kinetic Considerations: Principles, Commonalities, and Diversity of Gender- and Age-Related Metabolic Capacity

#### From Bioactive to Effect—The LADME Processes

**Figure 1:**
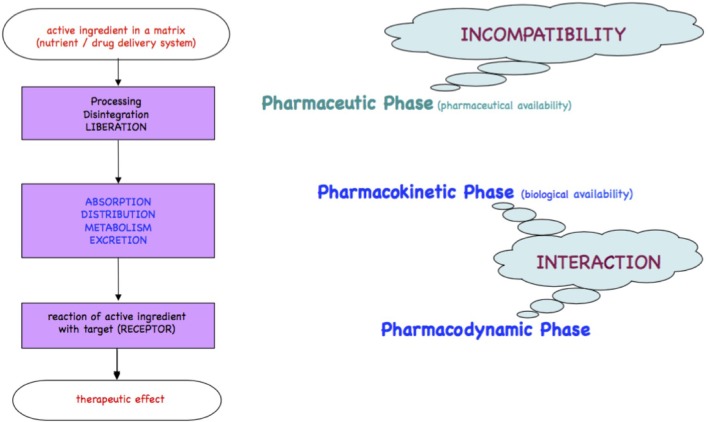
Ariens Scheme. A substrate freely accessible from a medicinal product or from foodstuff is orally taken and passes the pharmacokinetic LADME steps (liberation, absorption, distribution, metabolism, excretion). A part of the bioactive or xenobiotic may find in the pharmacodynamic step its targets such as receptors at cell surfaces, cell nuclei, or circulating metabolites to yield an effect.

Medicines or foods will be either readily incorporated if comparable to physiological substrates or metabolized for elimination as xenobiotics. The processes of the passage of xenobiotics across the intestinal wall, the body compartments and organs until receptors are found, are qualitatively described in the chronological kinetic steps Liberation, Absorption, Distribution, Metabolism, and Excretion and in the dynamic steps at the target/receptor as depicted in the Ariens Scheme [(10, 11); Figure 1].

To cope with variability in these processes genetic approaches have to be applied. Mutations on a single nucleotide (SNM, single nucleotide mutation) results in a genotype diversity and therefore different metabolic capacities, seen as polymorphisms in a population. Polymorphisms are a frequent reason for adverse drug events which increase in the case of polypharmacy. The interaction risk for a combination of 2 drugs is 13%, for 4 drugs 38%, and for 7 drugs 82% (12). In-patients receive 5–7 drugs. Thus, pharmacokinetic considerations are crucial. No estimation for food-food and food-drug interactions is available although they seem to occur even more frequent than drug-drug interactions. One of the reasons might be the lower severity of incidences. The higher probability of food—nutrient—drug interaction can be presumed by the vast amounts of food ingredients and the higher number of hepatic isoenzymes involved in this complex metabolism than in drug metabolism.

Ethnical groups show typical phenotypes. For instance, due to a mutation on the gene coding for the CYP2D6 isoenzyme in the Asian population, drugs being metabolized by this isoenzyme contain only 50% of the dose used in Europe. This mutation is important since many drugs (and possibly secondary nutrients) are metabolized by CYP2D6. For nutritional substrates less data and no interaction tables are available (13).

The objective of nutrigenomics research is to understand how nutrients influence gene and protein expression and why this expression varies between individuals. It is well-known that even though individuals were to eat similarly, some will become obese, others will develop cardiovascular diseases, or allergies. A recent project aiming at promoting research on interindividual differences has been adopted by the governments of COST member countries (COST action FA1401: “Interindividual variation in response to consumption of plant food bioactives and determinants involved (POSITIVe), https://www.cost.eu/actions/FA1403/#tabs|Name:overview)”^1^.

Evolution, globalization, and internationalization of nutritional habits make genetic impacts slowly disappear by genetic recombination and/or by adaption. Epidemiologic assessments are more and more difficult to interpret since at least in Western countries mixed populations lead to a recombination of genetic codes. Otherwise, as foodstuff consumed is not only produced in the consumers region as compared to times of regionally dominated markets, but is rather of international provenance containing new ingredients and components, genes do not only determine how food is metabolized, but food acts the other way around, i.e., by putting pressure on long-term genetic and medium-term epigenetic adaption. This will lead to new diagnostic tools such as genetic profiling and to novel interventions on an individual level (14–23).

#### Bioaccessibility and Liberation of Bioactives

Flavonoid-remnants of processed flavonoid-rich foods show various degradation patterns and quantities depending on the excess steam and heat applied. Tomato ingredients seem to be steam-volatile as Italian sugo for instance keeps more lutein, β-carotene, and lycopene than steamed tomatoes. Long heating processes lead to degradation of anthocyanidins in blueberries as only 3% remain after blueberry jam production. Precious Mungo sprouts components (genistein, daidzein) are fairly well-conserved by gentle roasting. Green tea macerated in 80°C water for 3 min keeps higher bioactive contents than when extracted at hotter temperatures (24).

#### Absorption and Distribution

Absorption of nutrients and xenobiotics is mainly an individualized procedure depending on a human being's metabolic capacity. This in turn varies with age, gender, ethnicity, and inherited metabolic performance. Gastric tube's morphology and physiological function shows major gender differences. In men the mean gastric fasting pH is 2.15 whereas it is situated between a range of 2.5 and 2.8 in women. This is a nearly 5-fold higher acidity in a male stomach and explains the higher prevalence of duodenal ulcers in men. A sufficiently acidic pH below 3.5 is determinant for the activation of pepsin from its precursor pepsinogen, duodenal secretin stimulation (at pH 4.0–4.5), and pancreatic enzymes' release as well. Gastric and intestinal liquid differ in volume (bigger in men), in emptying, in gastrin and bicarbonate concentration, and in colon transit times (shorter in men). Female hormone regulation by estrogens and progesterone is depressing gastrointestinal motility in women especially in pregnancy. Endocrine secretion in women is triggered when nutrients are provided and not permanently as compared to men's endocrine secretion and growth hormone release. Acidity at this upper GI tube is increasing from the fasting pH of 2–5 to pH 1.7 immediately after a meal intake and dropping to pH 4.3 toward the end of the digestion procedures after 2–3 h. As a summary, absorption is “quantitatively and qualitatively influenced by gastrointestinal pH and by intestinal motility” (12). On the other hand, absorption of precious nutrients is substantially decreased by tannins from tea or coffee due to precipitations (25).

For pepsin activation, which depends on a pH <3.0–3.5, and bactericidal effect, higher gastric pH of women would do as well. However, the gastrointestinal milieu changes fundamentally pH permanently to >4 both upon medication with antacids such as proton pump inhibitors (PPIs) and as a result of aging.

Drug or nutrient availability does not depend only on active carrier driven transports, but also on diffusion. Long-term PPI treatment switches the stomach's function almost permanently off. The underlying pH change has consequences for the solubility and therefore for the bioavailability of a important number of micronutrients such as vitamins C, B12, folate, and oligo-elements such as zinc, iron, magnesium, and calcium, and even of medicines such as ketoconazole, itraconazole, atazanvir, cefpodoxime, cinnarizine, enoxacin, dipyridamole, various oligoelements, or ascorbic acid, which are less bioavailable, and of nifedipine, digoxin, penicillin, erythromycin, or alendronate, which are more bioavailable (26–31).

Transport across the membranes and thus efficacy depend dramatically on gastrointestinal pH conditions. These transports may be bidirectional. Whether or not non-dissociated or dissociated substrates will be more readily absorbed depends on the absorption mechanism and the similarity to physiological molecules (passive diffusion, carrier-mediated transport, etc.). An important membrane-bound transporter at the apical side of enterocytes and at many other membranes, e.g., liver and blood-brain-barrier, is the P-glycoprotein (P-gp). P-gp is multi-drug-resistance1-Gene (MDR1) which builds an efflux of xenobiotics out into the lumen. It is often co-localized with CYP3A4,5,7 isoenzymes., For the P-gp substrates, just as for these isoenzymes, inhibitors and inducers can be distinguished (12, 25). pH is not only determinant for absorption of nutrients, but also for non-nutrient substrates, e.g., allergens and other xenobiotics such as non-steroidal-anti-inflammatory drugs (NSAIDs) or benzodiazepines. These xenobiotics have rather low pK_a_ values. Their absorption by passive diffusion therefore needs acidic pH conditions for absorption from the upper GI tube and treatments with antacids such as PPIs are likely to inhibit the quantitative absorption of NSAIDs or benzodiazepines and result in clearly lower anti-inflammatory effects (32).

Carbonates as antacids currently used in single doses beyond 1 g, “will have an onset of about 6 min and a time with pH values over 3.0 of about 2 12 h.” Ranitidine, meanwhile obsolete as H_2_-antagonist, was used for reflux in doses of 75 mg which led to an onset of 65 min. Its effect was rather unreliable and lasted for 36 min to 13 h (33). As a comparison, today's favorites and blockbusters pantoprazole and esomeprazole (both used in daily doses of 40 mg) yield a median 24 h pH of 3.7 vs. 4.7 in the steady state after 5 days. Long-term treatments with esomeprazol and pantoprazole have antacid effects of keeping gastric pH over 4 for 16.1 and 10.8 h, respectively. The long half-life times of these PPIs cannot be antagonized by any antidot. pH must in situations of need be imitated by ingestion of acidic beverages. Restoration *ad integrum* of the proton pump by *de novo* biosynthesis of the H^+^K^+^-exchanging ATPase will last several days. “The production half-life is approximately 50 h” (34–38).

Digestion-resistance during gastrointestinal transit is an important feature of food allergens. This resistance is supported by antacids, e.g., PPIs. A particular risk is arising for elderly patients suffering from gastroenterological diseases, tumors, and infections and consuming regularly “crustacean, eggs, fish, milk, peanuts, soybeans, tree nuts or fruits, and wheat.” In addition, with the loss of gastric acidity, the antibacterial intestinal activity becomes ineffective. Therefore, a net risk of “bacterial overgrowth of Clostridium difficile, Campylobacter jejuni, and Salmonella ssp.” is emerging. “This is also true for the inflammation, ulcer, and stomach cancer associated Helicobacter pylori which produces big amount of urease leading to a shift in gastric pH from between 2.2 and 2.8 to a range of 6–7” (39–42). There is a strong correlation between PPI treatments and “various pathologies such as lactose intolerance, celiac disease, atrophic gastritis, rheumatoid arthritis, and/or diabetes mellitus” (43–46). It is highly important to consider the consequences of pH increase on absorption of micronutrients, mainly iron, copper, and further co-factors of essential enzymatic metabolic reactions. Whereas, B-vitamins are co-enzymes in pyruvate decarboxylation and citric acid cycle, iron and copper are needed in the oxidative phosphorylation (respiratory chain) as part of some of the five enzyme complexes and as prosthetic group in hemoproteins.

Absorption of such oligo-elements follows the rules of solubility of aqua-complexes which is in turn dramatically depending on pH. The example of iron aqua-complexes shows that at pH conditions encountered at the jejuno-duodenal junction, where pH raises steeply, iron becomes less soluble as a result of a sequence of deprotonations of its aqua-complexes from a 3- or 2-fold positively charged to an uncharged insoluble complex in both cases of ferric iron and ferrous iron: (32, 47–49).

[Fe(H_2_O)_6_]^3+^ -> [Fe(H_2_O)_5_(OH)]^2+^ + H^+^ (pK_a_ 2.2)
[Fe(H_2_O)_5_(OH)]^2+^ -> [Fe(H_2_O)_4_(OH)_2_]^+^ + H^+^ (pK_a_ 3.5)
Fe(H_2_O)_4_(OH)_2_]^+^ -> [Fe(H_2_O)_3_(OH)_3_]^0^ + H^+^ (pK_a_ 6.0)
[Fe(H_2_O)_6_]^2+^ -> [Fe(H_2_O)_5_(OH)]^+^ + H^+^ (pK_a_ 6.74)
[Fe(H_2_O)_5_(OH)]^+^ -> [Fe(H_2_O)_4_(OH)_2_]^0^ + H^+^ (pK_a_ 9.5)

**Figure 2:**
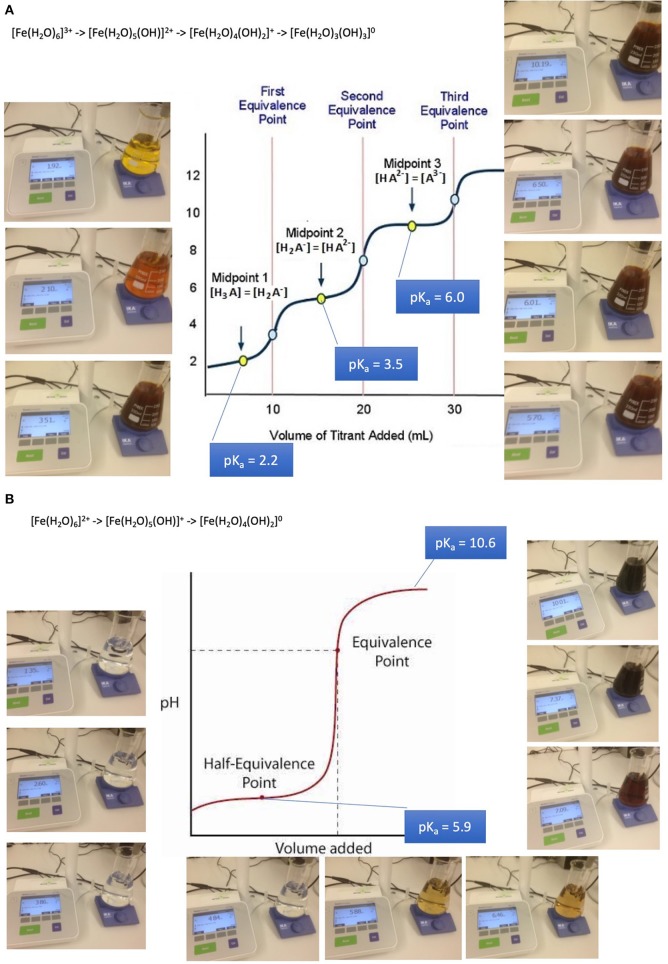
Absorption and bioavailability of iron. “Free” iron exists in aqueous solutions as aquo-complexes and is therefore an acid which deprotonates step by step. It solubility depends on its oxidation state and pH. **(A)** Precipitation of iron-(III)-aquo-complexes starts already at fairly acidic conditions. This leads to a loss of solubility decreasing from the acidic environment in the stomach and the proximal parts of the duodenum. An amorphous mass once precipitated cannot be absorbed from the GI tube an more. **(B)** Precipitation of iron-(II)-aquo-complexes are becoming critical only at neutral an alkaline conditions. Ferrous iron condensates only at such conditions. This is the reason why ferrous iron is absorbed more readily than ferric iron which is at these pH conditions already precipitated. Ferrous iron is therefore suitable for iron-anemia treatment.

These mechanisms lead to iron precipitates in the course of the passage along the GI tube starting to be relevant from about pH 3 upwards (Figures 2A,B). Precipitates are composed of colloidal and amorphous ferric iron hydroxides. Condensation is triggered by deletion of water out of the complexes and formation of hydroxo bridges. It is obvious that that such an amorphous mass cannot be absorbed from the GI tube.

Anemia caused by iron deficiency is treated more successfully by medicines containing ferrous iron Fe(II+) as an active ingredient, because the main loss of the alternative ferric iron would result close to the junction of duodenum to jejunum when within 10 cm distance alkaline conditions become predominant. Remaining ferric iron may be reduced in the distal duodenum to its ferrous form [Fe(H_2_O)_6_]^2+^. Ferrous condensates form only at alkaline conditions. This is the reason why ferrous iron is absorbed more readily than ferric iron which is at these pH conditions already precipitated and lost for absorption. It is wise to supply iron dissolved in fruit juice such as orange juice due to ascorbic acid contributing to the stabilization of better absorbable ferrous iron, increasing the absorbed amount up to 3-fold. The role of ascorbic acid contained in fruit juice is to enhance acidic conditions in the absorption region in case of PPI treatment and to reduced ferric to ferrous iron. The repeated supply of smaller amounts of oligoelements is more likely to lead to an efficient absorption and distribution than with high loading doses. Copper and cobalt but not manganese and zinc aquo-complexes suffer as well from partial loss when pH conditions are unfavorable for absorption (32, 47–49).

Depending on age, gender, physical activity and many more factors, the ratios of body compartments vary and therefore the distribution according to the hydrophilic—lipophilic profile of a substrate.

##### Ontogeny, peri- and post-natal phase, adaption, lactation

Availability of active pharmaceutical ingredients is particularly low in newborns as metabolizing enzymes in the liver are most active after birth and declining when age increases. Not only activity but also inducibility is highest in newborns. However, post-natal inducibility is lower than in the embryo stage. Peaks are observed before birth (50). For this stage of ontogeny and of neonatology, no information about a gender impact nor about ethnical variability is available.

Full peptic digestion capability develops after 2 years only, whereas antisepsis due to low pH in the stomach is immediately built up after birth: “In full-term babies, gastric acidity changes within 1 h from 6.1 to 5.4, within 2 h to 3.1, and within 6 h to 2.2. pH increases afterwards for 10 days and goes down again only slightly. Acid output similar to adults is reached by 24 weeks. pHs were measured either by potentiometry or by titration in older methods (51–54). Results should be equally reliable with either one of the methodologies of measurement. “Pre-term born babies have a gastric pH >4 for 80–90% of time, the acidity only rising with age” (55).

Bottle-fed babies have a similar gastric pH to breast-fed babies. “An increase in gastric pH to around 4.0 is observed for infants between 1 and 12 months with chronic diarrhea and protein malnutrition, combined with bacterial overgrowth essentially with Gram-negative bacilli, in 57% of the cases. The bacterial overgrowth happens at the moment of the evolution of the disease to a chronic state. Breast-fed babies do not have bacterial overgrowth of gram-negative bacilli and no diarrhea” (56). This bacterial overgrowth is favored by bottle-feeding with formula products, as immunoglobulins from lactating mothers and timely physiological pH adaption are lacking. In addition, children aged 4–36 months suffering from GERD (gastro-esophageal reflux disease) and treated with H_2_ antagonists or PPIs for 8 weeks showed a higher risk for acute gastroenteritis and pneumonia, although otherwise healthy. These risks persist after stopping the therapy (39).

##### Childhood

*Prepubertal childhood*. There is a gender difference in prepubertal oligo-element absorption: In the fasting state, male preadolescents absorb 35.2% and female preadolescents 45% of an iron loading dose. As supplement in a meal, preadolescent men absorb 14.8% and preadolescent women 24.7% of a dose. Serum ferritin differed only marginally in this study. As a result, iron anemia is as high as 12.1% in boys aged 11–15 and only 6.1% in girls of the same age (57).

*Pubertal childhood*. Hormonal biosynthesis and changes all over life is mirrored not only by body morphology, but also by the extra-hepatic regulation of metabolic processes. The breast consists of differently developed lobes and is equipped with the adequate CYP isoenzymes such as aromatase to locally biosynthesize steroids from circulating precursors. These hormones are liberated and systemically measurable. Estradiol and progesterone bind to their receptors ERα, ERβ, PR A, and PR B and initiate the cell signaling cascade finishing up in the cell nucleus. Estrogens induce the ductal development, progesterone the lobes and alveolus. Type 1 (65–80%) and type 2 lobes (10–35%) predominate in child-less women. Type 3 dominates (70–90%) in women after delivery. In the menopause, involution occurs, and type 1 dominates again. Risks for breast cancer are an early menarche, a late menopause, and a higher age at the first pregnancy. A protection is evident from an early pregnancy and delivery (58).

##### Adulthood

*Ovulation-suppressed women*. Apart from iron, another important co-factor of enzymes is copper. In the age group of 20–59 years old persons, its absorption is generally higher in women (71%) than in men (64%). This difference disappears later in life at the age between 60 and 83 years. Copper and ceruloplasmin plasma levels of women aged between 20 and 39 years taking ovulation suppressors tend to be higher than in women with physiological cycles (59).

Estradiol suffers from an extensive hepatic first-pass effect. As a result of its low bioavailability of <10%, its derivative Ethinylestradiol is the most widely used estrogenic component of combined oral contraceptives. The absorption process differs individually in length (1–2 h), extent (generally around 90% bioavailability), and the time for reaching the maximum peak of blood level (1–2 h mostly, with observed lag times of up to 6 h). Steroid concentration in the portal vein blood after absorption and hepatic first-pass is still 25–65% of the amount ingested and has an effect on several globulins such as thyroid binding, sex hormone binding, corticosteroid-binding globulins, angiotensinogen, as well as coagulation and fibrinolytic factors. 2-hydroxylations by CYP3A4 and CYP2C9 dominate with wide individual variation, whereas 4-, 6-, 16α-, and 16β-hydroxylations are compromised to physiological estradiol. Besides glucuronidation, sulfate-conjugates at positions 3 and 17 are readily built and are 10-fold more concentrated in the circulation than ethinylestradiol itself. Partially, to 11 and 21%, respectively, the sulfates are deconjugated to ethinylestradiol during enterohepatic recirculation. Plasma levels of ethinylestradiol differ markedly between ethnic groups. Highest plasma levels were found in Nigerian women, lowest in women from Thailand. Nigerian women excreted mainly conjugates of ethinylestradiol and hardly any oxidation derivatives (58).

Progestins used for contraception and/or post-menopausal hormone replacement therapy vary widely in structure, absorption and metabolism. Large inter- and intrasubject variability is observed. The C_max_ is reached within 1–3 h. Plasma levels are between 70% and more than 90%. T_1/2_ differs from between 8 and 12 h for norethindrone and dienogest to between 50 and 80 h for cyproterone acetate (60).

Obesity is considered an inflammatory disease with elevated levels of some cytokines such as IL-6 and TNF. Cytokines are known as transcriptional regulators of the CYP450 expression. In addition, fatty livers might affect the function of hepatic isoenzymes. A decrease of activity is reported for CYP3A4,5,7 and CYP2E1, whereas the efflux transporter P-gp is expressed higher. Although CYP450 isoenzymes' activity decreases and conjugation increases, the “one dose fits all” approach is applicable in both obese and normal weight women, as oral contraceptives influence the hypothalamic—pituitary—ovarian axis and the feedback loops similarly with a tendency toward more follicle maturation in obese women (60). A higher part of hormonal contraceptives is distributed toward the fat mass in obese subjects. This bears a higher risk of hormonal contraceptives failure as contraceptives' plasma levels decrease. However, no difference in distribution volumes of ethinylestradiol and levonorgestrel between normal and obese BMI subjects could be demonstrated. Instead, the time to reach the levonorgestrel steady-state was found to be adversely affected by obesity and the half-life in obese subjects was twice as long. While peak levels of oral contraceptives (ethinyl estradiol and levonorgestrel) are somewhat lower in obese women (BMI 30.0–39.9) compared to normal weight women (BMI 19.0–24.9), the through levels over the cycle are similar. The small pharmacokinetic differences do not activate ovarian follicles (61).

*Pregnancy*. “As a result of hormonal changes, pregnant women often suffer from heartburn induced by reduced esophageal sphincter pressure and heartburn due to sex hormones, essentially progesterone” (62). “In this patient group, short-term acid neutralizers or H_2_-antagonists should be preferred instead of PPIs in possibly meal-free phases in order to omit the risk of incomplete protein digestion and the risk of predisposition to immune responses of the child. This risk is indeed confirmed in a Swedish study correlating the incidence of allergy and asthma of babies with anti-ulcer consumption of their mothers during pregnancy.” The allergy incidence of the discharged children was 5.03%. This significance was shown by an odds-ratio (OR) of 1.43 with a 95% confidence interval (1.29–1.59). The development of childhood asthma is higher in exposed children (5.6%) than in the population (3.7%), statistically validated by OR 1.51, 95% CI 1.35–1.69 (63).

#### Metabolism

To cope with food-derived interaction variability, all 57 CYP450 isoenzymes should be assessed. In addition to pharmacotherapeutically relevant CYP450 isoenzymes such as those listed in the Flockhart tables (64), substrates, inhibitors and inducers originating from food, e.g., anthocyanidins, flavonoids, vitamins A, B1, B2, D, E, brassica species (broccoli, Brussel sprouts, cauliflower), chargrilled meat, tobacco, ethanol, St. John's wort, grapefruit juice, or Cassia (Chinese) cinnamon, and many more have to be of interest to nutritionists and nutritional medicine. In clinical nutrition, not only deficits are challenging, but too much intake as well. Well-known to endocrinologists were the goiter inducing brassica factors of mustard oil glycosides from brassica species. This effect was triggered by thiocyanate liberated and competing with iodine for the thyroid peroxidase catalyzed iodination of thyroglobulin (65).

Metabolic reactions are predominantly assumed by the cytochrome P450 superfamily. P450s are present in Homo Sapiens, and over 4500 individual members are known in all living species. “450” relates to the Soret region in the absorption spectra of these enzymes where their λ_max_ are located. CYP450s are classified into 18 families and 43 subfamilies. Apart from gene expression and the phenomenon of polymorphism, gender and age have an impact on the activity and thus on inter-individual variation. Members of the families CYP1, CYP2, and CYP3 are polyvalent and therefore suitable for metabolic transformation of a large number of drugs and structurally diverse xenobiotics, whereas the families CYP11, CYP17, CYP19, and CYP21 tend to have only a few substrates, predominantly their physiological substrates, i.e., steroid hormones, and to be therefore highly specific (66). This enzymatic polyvalence is clinically important mainly for toxicological reasons. However, these enzymes may be saturated and become rate-limiting pacemakers. Substrates may accumulate and adverse side effects may arise from increasing blood levels of the substrates. Inhibition may be established within a few days. Induction develops more slowly, in general within some weeks (12).

Metabolism is discussed mainly in terms of the hepatic part due to its predominance, although it happens also in extra hepatic hormone dependent tissues, e.g., CYP2S1 in epithelial tissues, mainly skin, but also kidneys, intestines, lungs, nasal epithelium. This is the reason why efficacy of pharmacotherapy may depend on target tissues, e.g., retinoids in skin. Hepatic and intestinal P-gp and other transporters are expressed to a higher degree in men than in women. In men, some isoenzymes have a higher activity, due to induction, e.g., CYP1A2, CYP2C9, CYP2E1. CYP1A2 can be induced to be 40 times as active in case of a high charcoal-grilled meat consumption. In women, some CYP450 isoenzymes are more expressed than in men, e.g., CYP3A4,5,7 as a function of the menstrual cycle with top level before ovulation and in pregnancy, CYP2A6, CYP2B6, or CXP2D6, which is highly expressed only in the fertile phase, CYP3A4,5,7.

Inconsistent data seem to arise from cross-influences of P-gp and CYP3A4,5,7. The efflux glycoprotein and the CYP450 isoenzymes are co-localized. Thus, in clinical studies evaluating both transporting P-gp and metabolizing CYP3A4,5,7 care has to be taken to omit confounding (25). Research on nutrients in this domain is scarce and remains to be established. The reason might be a lack of using histological and cell-biological methodologies in nutrition research as compared to *in vitro* human liver models currently used in pharmacological and pathological research (11).

##### Biochemistry of cytochromes P450

**Figure 3:**
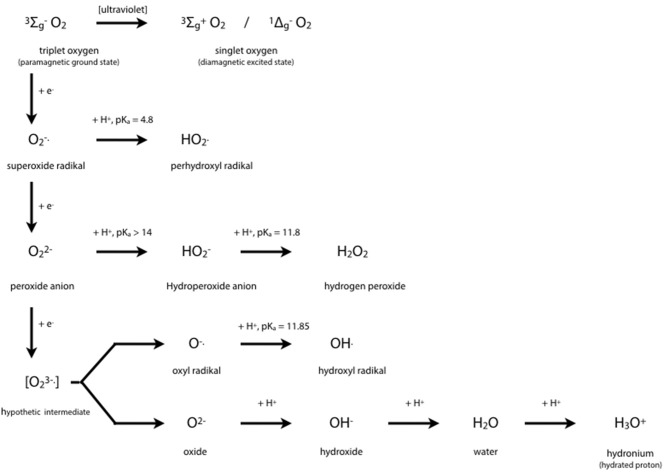
Oxygen Chemistry. Dioxygen itself exists in its ground state as biradical. This is the reason why only the existing partial pressure is healthy to mankind and hyperbaric atmosphere would be toxic. Free radicals derived from successive 1-e^−^ reductions of dioxygen may be harmful to many organic molecules at physiological conditions. Oxygen derivatives are indispensable intermediates in oxidoreductase reactions such as detoxifications, hydroxylations, peroxidations.

Cytochrome P450 isoenzymes are tetramethylated hemoproteins, such as cytochrome c, ferriprotoporphyrin IX, hemoglobin, or myoglobin, with iron (+III) as central atom, and widely distributed throughout the biological world. From 1980, CYP450's crystal structures were solved for 54 unique structures (67). Subtle changes from one molecule to a closely related one generally lead to drastic changes in behavior, i.e., to different groups of hemoproteins such as monoxygenases (= mixed-function oxygenases), peroxidases, peroxygenases, and oxygen carriers. Typical enzymatic cycles and intermediate compounds involved provide highly reduced oxygen derivatives as an oxidant (Figure 3). However, CYP450 isoenzymes do not only act as monooxygenase to detoxify. Today's knowledge hints at a more complex role in regulation on hepatic and extra-hepatic tissues.

The central iron atom of hemoproteins carries oxygen in different oxidation states, i.e., as reactive oxygen species which either oxidize substrates of the enzymes compounds I and II by 1 e^−^ or 2 e^−^ donations, respectively, or contribute to irreversible enzyme inactivation in case of excess substrate by the enzymes compound III. Inactivation of the enzyme occurs by cleaving the heme moiety and liberating iron which in turn contributes to Fenton-like reactions producing highly oxidative hydrogen radicals:

Fe^2+^ + H_2_O_2_ → Fe^3+^ + OH^.^ + OH^−^

Copper may substitute for iron. It yields an even faster production of hydroxyl radical (65).

**Figure 4:**
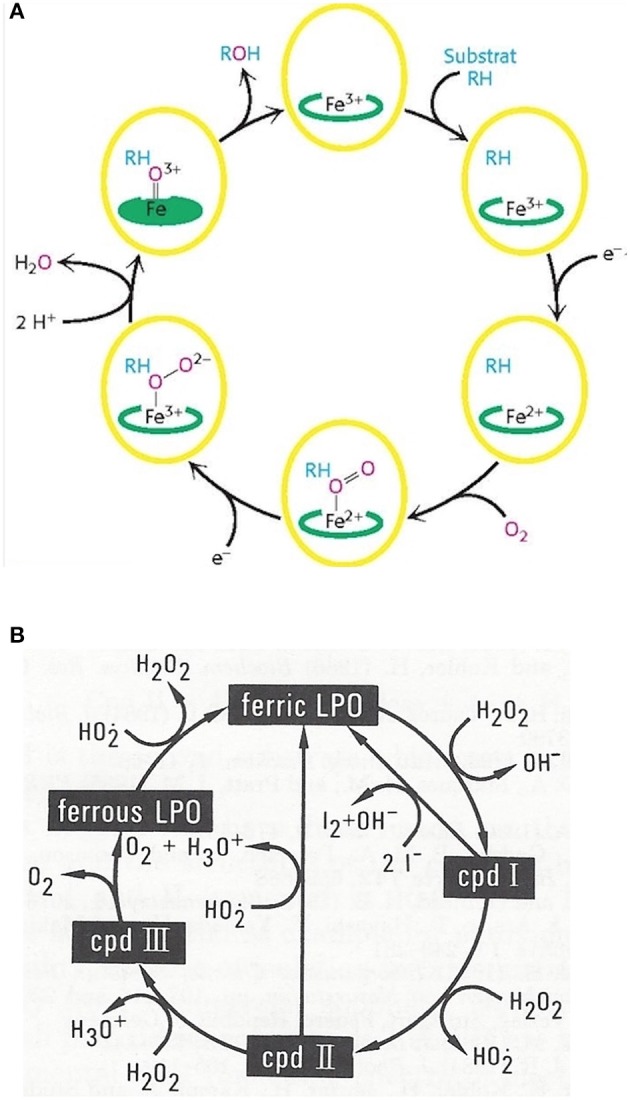
Outline of the catalytic cycles of oxidoreductases. **(A)** Hydroxylation of substrates of aromatase requires the activation of oxygen. This activation is promoted by the heme prosthetic porphyrin ring system. An electron transfer reduces the ferric to the ferrous intermediate state, which enables iron to bind dioxygen. Another electron transfer induces a rearrangement of the highly electron-dense π-system and cleavage of the O-O bond. One of the oxygens is protonated and liberated as water. The remaining oxygen is configured as ferryl intermediate which extracts a hydrogen atom from the substrate. A transient radical of the substrate thus formed captures the OH group from the enzyme intermediate to end up as hydroxylated substrate. With the permission of Berg et al. (68). **(B)** Pathways in lactoperoxidase-catalyzed H_2_O_2_ metabolism. I^−^ reacts with compound I by direct 2e^−^ transfer. The normal peroxidatic cycle includes ferric lactoperoxidase -> compound I -> compound II -> ferric lactoperoxidase. H_2_O_2_ in excess leads to formation of compound III and reconversion to the resting enzyme via the ferrous state. Compound III pathway is combined with irreversible inactivation of the enzyme. The conceivable structures of the heme moieties of lactoperoxidase's ground state and intermediate enzymatic compounds show a movement of iron out of plane (not depicted) (65, 69).

Monooxygenation require NADHP and O_2_ (Figure 4A):

RH + O_2_ + NADPH + H^+^ → ROH + H_2_O + NADP^+^

To bind dioxygen, the prosthetic group must be reduced from the ferric to the ferrous state by a first 1-e^−^ transfer from NADPH. Another 1-e^−^ transfer from NADPH induces a structural rearrangement of the high electron-density on the π-system and the shift of the density on the dioxygen which leads to O-O bond cleavage. One of the oxygens is liberated and protonated to form water. The remaining oxygen is configured as highly reactive ferryl intermediate (Fe = O). This ferryl intermediate obtains a hydrogen atom from the substrate RH. After the hydrogen release the substrates forms a radical intermediate which in turn captures the preformed hydroxyl group from the enzyme intermediate to form the hydroxylated substrate. The release of OH brings iron back to the ferric state (68). Thus, one of the oxygen of dioxygen will end up as hydroxyl function in the substrate. The second oxygen atom will be reduced to water.

Mono-oxygenation can be performed by a peroxide shunt as an alternative. Single oxygen donation from substrates such as peroxides lead to the shortly living and hardly detectable π-cation radical compound I. This shunt resembles to a peroxidative cycle as depicted for lactoperoxidase (LPO) (Figure 4B). In peroxidases, the catalytic cycle of P450 isoenzymes starts on binding the substrate, seen as a spectral change. e^−^ source is NAD(P)H via cytochrome P450 reductase. The reduction equivalent of iron is situated mainly paired in d-orbitals to yield the low spin ferrous state. Molecular dioxygen is bound covalently. Another e^−^ transfer reduces the dioxygen adduct, which is rapidly twice protonated, and liberates water. The resulting intermediate correlates to the highly reactive π -cation radical compound I which catalyzes any further reaction, e.g., a hydroxylation. From compound I upon release of the product, the enzyme reconverts to the resting state (70). The normal peroxidatic cycle includes ferric lactoperoxidase -> compound I -> compound II -> ferric lactoperoxidase. H_2_O_2_ in excess leads to formation of compound III and reconversion to the resting enzyme via the ferrous state. Compound III pathway is combined with irreversible inactivation of the enzyme (69). The heme moieties of lactoperoxidase's resting ground state and of its intermediate compounds are characterized by the conceivable movement of iron out of plane which impacts solubility and reactivity (65).

#### Excretion

As absorption and excretion sum up to a typical Bateman-shaped curve representing the plasma level of a single dose, there is a complex interrelation and regulation of biochemical pathways. Isolated views lead to erroneous interpretations of pathophysiological situations. A typical example is the assessment of the impact of salt intake on the highly public-health-relevant hypertension. Both excess and insufficient supply of sodium are recognized as inductors of non-communicable diseases. However, this does not yet qualify only intake nor excretion as the only reasons of hypertension. The question is rather what comes first, overconsumption of unhealthy foodstuff as inductor of cardio-vascular deterioration followed by manifest diseases, or rather the other way around, i.e., heart and kidney insufficiencies followed by the inability to excrete sodium efficiently. So far, not enough evidence has been presented for neither one of the two theses. It is likely that individual histories differ. Corresponding clinical studies suffer almost generally from systematic errors such as biases and confounding factors as well as unprecise exposure and outcome conditions. A study claiming that BMI is the main contributory modifiable factor of blood pressure level included in a meta-analysis 8,670 volunteers from a network-questionnaire on age-adjusted associations of hypertension and life-style. The questionnaire included a 3 times 24 h recall of dietary intakes. The outcome was a claim of hypertension being associated with elevated BMI, age, and education and of salt not being associated with systolic blood pressure in either sex after multiple adjustments (71). Another study did not find a hypertension lowering effect of low sodium intake in a particular regional study (72). The contrary claim that salt reduction lowers blood pressure was issued as a result of a meta-analysis including 3,220 participants in 34 trials. These participants reduced their daily salt consumption to 4.4 g for 4 weeks. The results reported and interpreted included a reduction of systolic blood pressure of −4.2 to −2.1 mm Hg over all, of −5.4 to −2.8 mm Hg in hypertensive participants, and of −2.4 to −1.0 mm Hg in normotonic participants (73). These marginal changes do not seem to be highly reliable. They contribute to implausible results in human nutrition research (4, 7). It is advisable to issue no claim at all actually due to insufficient proofs from both observational and experimental studies (74):

- Blood pressure changes reported are modest and variable
- Effect seems to be more distinct in the elderly and in subjects suffering from higher blood pressures
- There is no evidence that a salt intake reduction is safe or cardioprotective
- There is no comparison of cardioprotective, safe, efficacious, and economical medicines indicated in hypertension
- There is no convincing outcome assessment focused on the length and quality of life that would justify a public health recommendation to a whole population rather than to a selection of individuals in the context of personalized nutritional medicine

### Pharmaco- and Nutrient-Dynamic Considerations

#### Steroid Metabolism, Aromatase, Estradiol Receptors, and Breast Cancer

**Figure 5:**
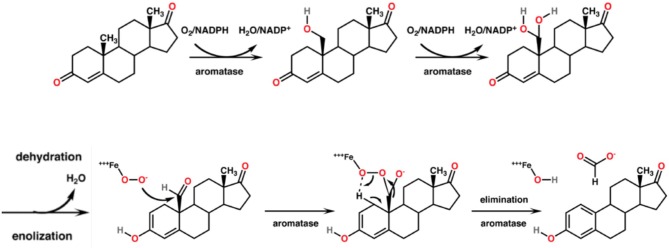
Mechanism of the aromatase-catalyzed enzymatic biosynthesis of 17β-estradiol from its androgenic precursor testosterone. The α,β-unsaturated ketone of ring A of the steroid is transformed to a phenolic structure. The biosynthesis consists of three consecutive reactions, two hydroxylations followed by a C-C bond cleavage.

CYP19A1, syn. Aromatase, is the CYP450 isoenzyme catalyzing the transformation of testosterone and androstenedione to estrone and estradiol. The enzymatic reaction consists of a concomitant demethylation and sterol ring A aromatization starting with two conventional hydroxylations yielding the 19-hydroxymethyl derivative of the substrate and a 19-gem-diol, which rapidly decays to an aldehyde. This one is cleaved at the C-C bond at C19 yielding formic acid which has incorporated both oxygens enzymatically from molecular oxygen (75). The monooxygenation is depending on the activation of molecular oxygen in CYP450 isoenzymes [(76); Figure 5]. The catalytic site *in vivo* consists of iron chelated as porphyrin derivatives. Nitrogen compounds replacing porphyrin as chelator are interesting candidates in chemical *in vitro* research as reaction kinetics differ markedly. Such candidates are deferiprone, deferoxamine, deferasirox (77).

The main targets of medicines indicated in breast cancer are aromatase (by aromatase inhibitors, e.g., tamoxifen) and selective estrogen receptors (modulated by selective estrogen receptor modulators SERMs). 17β-estradiol (E2) is the most biologically potent estrogen in breast tissue. However, breast cancer and highest E2 blood levels are not directly correlated. Most women develop breast cancer after the menopause. In this phase of life, remaining E2 plasma levels are 10 times lower due to ovarian exhaustion. Thus, the uptake of E2 from ovarian biosynthesis via blood stream into the mammary gland is unlikely to be responsible for local estrogen concentrations in breast tumors. Another mechanism must be the causation of neoplasms. It is more likely that “hot spots” of aromatase become highly active to increase the intra-tumor biosynthesis of estrogens from the precursors testosterone and androstendione circulating in the plasma (58, 78). Thus, the neoplasm itself induces a local production of aromatase which increases in turn the effectiveness of therapeutically and preventively used aromatase inhibitors. Apart from CYP19A1, a mixed-function monooxygenase, tumorigenesis has been explained as an effect of lactoperoxidase present in the mammary glands, a peroxidase, oxidizing estrogens at the 3-hydroxylgroup to free radicals (79).

**Figure 6:**
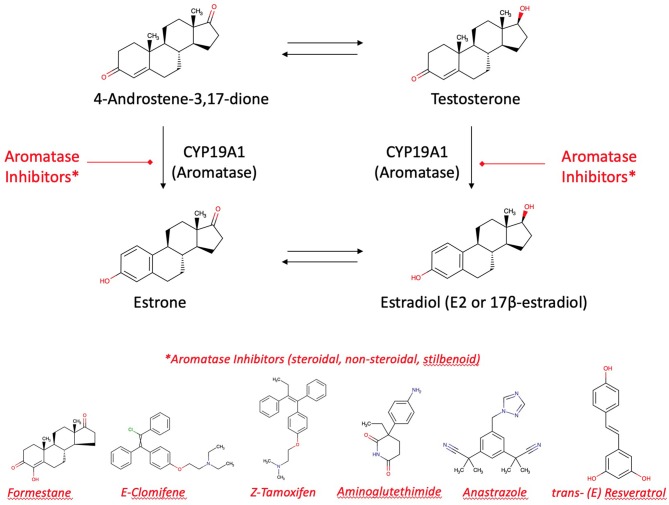
Aromatase and aromatase inhibitors. Estradiol and estrone are enzymatically biosynthesized by aromatase (CYP19A1) from androgenic precursors. Aromatase inhibition is the current pharmacotherapy and suitable prevention of breast cancer. There are more than 30 enzymatic reactions upstream, downstream, and sidestream of the aromatase reactions. Therefore, an delicate equilibrium in the hormonal status is highly regulated and fine-tuned.

Aromatase inhibitors are the therapy of choice for most post-menopausal breast cancer patients. It may be accompanied by an adjuvant therapy with aromatase inhibitors from plants. Alone, these aromatase inhibitors from plants are suitable to contribute for long-term breast cancer prevention [(13, 24); Figure 6]. However, enhancement or reduction of the enzyme activity or its protein expression by food ingredients is an understudied issue. There are a couple of structure requirements substrates should fulfill in order to interact with aromatase to exert an activity, either an effect or an inhibition (77, 80):

- α-/β-unsaturated ketone function
- This provides a flat configuration. Therefore, an aromatic ring system is also suitable
- Z-conformation at the double bond
- An asymmetric center (^*^C, spiro, chock, or ansa configuration in position γ to the ketone) wearing a methyl substituent. Testosterone is according to IUPAC rules (1S,2R,10R,11S,14S,15S)-14-hydroxy-2,15-dimethyltetracyclo[8.7.0.0^∧^{2,7}0.0^∧^{11,15}]heptadec-6-en-5-on
- Steroid binding site located on the apoprotein
- The aromatase substrate must fit into this 3D-structure.

#### Flavonoids as Inhibitors of Estradiol's Action

Aromatase is a target of flavonoid bioactives. Flavonoids' 3-dimensional partial structure ressembles the physiological substrates of aromatase, i.e., testosterone and androstenedione. Flavonoids compete therefore for aromatase and enhance or inhibit modulators of 17ß-estradiol receptors ERα and ERβ. The inhibitory effect on the enzyme needs a structural relationship between androstendione or testosterone and a partial structure of flavonoids (Figure 6). As a result, inhibitors and natural substrates compete for the enzyme and antagonize the transformation of the physiological substrates to estrogens. The mechanism of action of flavonoids is currently described as antioxidative effect. In fact, as the oxygen cannot be transferred from the enzyme's intermediate compound to the substrate but to the competing flavonoid, this explanation is in line with the competitive antagonism explanation and the classical pharmacological interaction concept.

Aromatase inhibitor stereochemistry and quantitative conditions at the target are essential to determine the effect it will have on the development of breast cancer. When using aromatase inhibitors, estrogen levels decrease in the plasma and locally in breast “hot spots,” decreasing the outbreak or progression of breast cancer. In addition to the pharmacochemical aromatase inhibitors, there are many natural components that can perform the same action, eventually with fewer adverse effects (81).

Inhibitory effects on aromatase have been described for many plants, mostly containing polyphenols/flavonoids [(82–84); Table 1]. The most popular plants are:

- (Green) tea and soy intake as a result of the active principles caffein, calcitriol, ellagitannins, flavonoids/polyphenol/isoflavones
- Buckwheat, millet, or brown and purple rice
- Cabbage, broccoli, cauliflower, kale, and bock choy
- Garlic and tomatoes as a result of indole-3 carbinol and carotinoid (lycopin) content
- Mangosteen, grapefruits, and other citrus fruits as a result of monoterpenes content
- Grapeseed due to procyanidin B dimer and resveratrol
- Pomegranates due to ellagitannins (the most potent being urolithin B)
- And mushrooms (white button, baby button, shiitake, portobello, and crimini) due to conjugated linoleic acid.

**Table 1:** CYP19A1 active food ingredients, their targets, and effect on the enzyme: Substrate (S), Inhibitor (–), Inducer (+).

**Plant and active ingredients**	**Targets**	**Effect on aromatase**
Allium species, containing allicin, S-allyl cysteine, S-allyl mercaptocystein, organosulfides (diallyl mono-, di-, trisulfide) • A. sativum = Garlic • A. ursinum = Wild garlic	Cell signaling Glutathiontransferase Apoptosis	–
Brassica species [Cruciferous vegetables (mainly sprouts)], containing indole-3 carbinol, organosulfides, sulpharophanes, isothiocyanate from glucosinolates • Broccoli • Brussels sprouts • Cauliflower • Kale and bock choy	Aromatase Cell signaling Glutathiontransferase Angiogenesis VEGF receptor NF-κ B Apoptosis	–
Citrus spp., containing prolin, betain, naringenin, monoterpenes, D-limonene • Grapefruit • Orange • Mineola	Aromatase COX-2 Cell signaling	–
Coffee, containing melatonin, and melanoidins	Aromatase COX-2 Cell signaling Metabolic regulation	–
Curcuma domestica and C. longa (Turmeric), containing curcumin	COX-2 ROS scavenging	–
Fruits (pigmented) and berries, containing ellagitannin, ellagic acid, sitosterol, anthocyanidines (delphinidine, myrtillin), phenolic acids • Urolithin B in pomegranate, • Rubus ssp.: lambertianin C in blackberries, sanguinin H-6 in raspberries, myrtillin in blackcurrant pomace • Fragaria × ananassa Duch.: agrimoniin)	Aromatase COX-2 Cell signaling Glutathiontransferase Angiogenesis/VEGF NF-κ B Apoptose	–
Ethanol	Aromatase	+
Fatty acids from animal or vegetable oils • ω-3 fatty acids ° docosahexaenoic acid ° eicosapentaenoic acid • ω-6 fatty acids (linoleic acid) • Fish-oil	Nuclear receptor Anti-oxidation	–
Grape seed, containing resveratrol	Aromatase COX-2 ROS scavenging	–
Green Tea, containing (–)-epigallocatechingallate	Aromatase	–
Honey, sugarcane molasses containing melatonin and melanoidins	Aromatase COX-2 Cell signaling Metabolic regulation	–
Liquorice (Glycyrrhiza glabra), containing glycyrrhizin, Glycyrrhic acid	Aromatase	+
Mushrooms • Agaricus bisporus (= white or brown button, baby button, portobello, crimini, champignon de Paris)	Aromatase	–
• Lentinula edodes = Agaricus edodes (= shiitaki, Chinese black mushroom, golden oak mushroom)		
Nuts, containing fatty and amino acids (e.g., arginine) • Walnut • Hazelnut • Cashew	Nuclear receptor VEGF receptor	–
Soy, containing phytoestrogens, stilbenes, and genistein	Aromatase	–
Testosterone (and Androstenedione)	Aromatase	S
Ubiquitary occurrence: Flavonoids. Polyphenols • Flavonols (kaempferol, rutin, myricetin, quercetin, spirenoside, galangin, rhamnetin) • Flavonones (hesperitin, naringin, naringenin, hesperidin) • Flavones (apigenin, flavone, luteolin, chrysin, diosmetin, diosmin) • Flavonolols (silibinin, silymarin, taxifolin) • Flavan-3-ols (catechin) • Isoflavones (genistein)	Aromatase COX-2 Anti-oxidation	–
Vitamins A, C, E, and provitamins, e.g., lycopene (= Ψ,Ψ-carotene), beta-carotene, xanthophylls, tomatine, solanin • Tomato (Solanum lycopersicum) • Watermelon • Cantaloupe • Pink grapefruit • Papaya • Apricot • Carrots • Banana	Aromatase COX-2 Cell signaling	–
Wholegrain, containing fiber, polyphenols, fatty, and amino acids • Buckwheat • Millet • Rye bread • Brown rice • Purple rice	Aromatase Nuclear receptor VEGF receptor COX-2	–

*For references see text*.

Induction of aromatase and thus increase of breast cancer risk can be associated with ethanol. Liquorice (glycyrrhizin from glycyrrhiza glabra) reduces serum testosterone and induces ovulation by induction of aromatase (85–90).

Various flavonoids have been tested and the effect been quantified on cell lines only, but not *in vivo* (e.g., H295R adrenocortical carcinoma cells). Chalcones and flavanones such as naringenin and hesperetin, flavones such as rotenone or luteolin, catechins such as theaflavin or stilbenoids such as resveratrol are stronger than other flavonoid derivatives (81, 91–94). Prophylaxis of breast cancer by flavonoid intake depends on the amounts consumed. Also, a protein-rich diet including 300 g soy protein corresponds to more than 100 g isoflavones per day is having an effect in breast cancer prevention. Although the potency of isoflavones is a mere permille of physiological 17β-estradiol, it may significantly modulate estradiol effects at the estradiol receptors due to its high affinity for the α-receptor subtype, inducing a competitive inhibition in this way. The relative concentrations of estradiol and of isoflavones competing for the receptor will determine the long-term prophylactic success (95).

Some authors describe more precisely the presumed mechanism of action of active ingredients. Purple rice extract inhibit VEGF (vascular endothelial growth factor)-induced angiogenesis at the cell surface receptor (96). Organosulfur compounds from garlic and other allium species are able to suppress cancer cell proliferation by inhibiting the cell division cycle of phosphatases rather than aromatase (97). 36 ß-caryophyllene oxide's target is the cell signaling cascade. Cell growth is inhibited and apoptosis induced by the suppression of PI3K (phosphatidylinositol 3 kinase) pathways and ROS (reactive oxygen species)-mediated MAPKs (mitogen-activated protein kinases) activation (98).

A further explanation for the inhibiting effect of flavonoids on aromatase is the inhibition of activity and expression of histone deacetylation. As acetylated histones widen DNA and therefore favor transcription, this mechanism corresponds to a procedure of switching genes on. Acetylation opens chromatin and enables transcription. Therefore, inhibition of acetylation might limit DNA replication in tumor tissues, an important regulation of repressing cancer genes. Switching off genes would be accomplished by silent (condensed) chromatin, enhanced by methylation of cytosines and deacetylation of histones. These switching genes on/switching genes off mechanisms are highly important for epigenetic labeling and adaption to changing environmental conditions (99–101).

Epigenetics also seem to play an important role in these interindividual differences. A study with 300'000 recruits from the Dutch army showed that famine has a statistically significant influence and that a fetal memory exists. Malnutrition during the first trimester of pregnancy experienced by the mothers of these recruits in 1944-45 (“hunger winter”) led to an elevated rate of obesity of their sons by the age of 19. The cause was a modification of methylation on the IGF2 gene which promotes growth during pregnancy (102–104).

Traditional antioxidation diet is one of the most approved explanation of inhibition of aromatase by phytonutrients of flavonoid derivatives. However, this means as well that flavonoids compete for the oxygen in the course of enzymatic oxidoreduction, and therefore of aromatase-catalyzed conversion of steroid precursors to estrogens.

#### Resveratrol

From resveratrol the two enantiomeres trans-(E)-resveratrol and cis-(Z)-resveratrol exist. Due to the (E)-enantiomere's stilbenoid structure resembling to a partial structure of α,β-unsaturated ketone of the steroid's ring A, aromatase is inhibited competitively by resveratrol (Figure 6). The trans-isomer is more stable. Trans to cis somerization is facilitated by UV light of 200–400 nm and high pH. Cis to trans conversion is facilitated by visible light of 400–800 nm, high temperature, and low pH. Roasting has a diminishing effect on polyphenolic composition and antioxidant activity. Sources of resveratrol comprise (105–113):

- Fresh grape skin: 50–100 μg/g
- Red wine: (98–1,803 μg/g cis-resveratrol, 0.5–26 μg/g trans-resveratrol)
- Peanuts skin: 0.02–1.79 μg/g
- Strawberry: 0.83 μg/g
- Hop: 0.23–2.28 μg/g
- Common buckwheat: 0.98–1.68 μg/g
- Tartary buckwheat: 3.43–3.5 μg/g.

#### Nutrigenomics and Further OMICS Technologies

The human body encodes approximately 50 members of nuclear hormone receptors on the DNA, consisting of DNA-binding and ligand binding domains. A number of cysteine residues binds zinc ions to stabilize the structure of these zinc-finger domains of steroid receptors. The impact of mutations on the genetic coding, currently known as single nucleotide polymorphisms (SNPs), is a key issue to prevent mistranscription and mistranslation (e.g., “the sun is now red” ≠ “the sun is not red”). Genomics, transcriptomics, proteomics, metabolomics, and other omics technologies have been identified as key technologies to find answers to unresolved questions such as to why individuals respond differently to equal bioactives^1^. During aromatase and SERM treatment, “either *de novo* or acquired resistance is seen with many patients. With *de novo* resistance, due to reduced influence of estrogen receptors (ER) activity in these types of tumors, there is no response to a drug upon initial treatment. Furthermore, some patients acquire resistance after initially responding to a drug treatment while still maintaining ER expression. This strongly suggests that ER may still play an important role after acquiring resistance to anti-hormonal drugs has developed.” Reasons for resistance comprise (77):

- Mutations and/or variants of estrogen receptors and of aromatase
- Modifications of the apoprotein of aromatase in its post-transcriptional or post-translational biosynthesis
- SNPs of enzymes involved in aromatase's prosthetic group biosynthesis, i.e., heme biosynthesis
- Modifications in the cell-signaling pathways triggered by docking substrates at the estrogen receptor.

Endocrine resistance is not yet fully understood. More research is needed in order to develop target specific drugs not affected by endocrine resistance. Apart from inborn errors in direct biosynthesis pathways, the all enzymes involved in the underlying biosynthesis pathways (up to multiple levels upstream) such as the porphyrin biosynthesis are important elements of an overall functioning metabolism. The ALA-synthetase catalyzed formation of δ-aminolevulinic acid, starting from succinate and glycine, is the first and rate-limiting step of heme biosynthesis. There is a particular role of the vitamin b12—tetrahydrofolate—methionine/homocysteine system as carriers of methyl bricks. The need of these carriers for heme biosynthesis explains why not every anemia is an iron anemia and therefore cannot be treated by iron supplementation. It may be diagnosed as pernicious anemia due to a vitamin b12 deficiency. Iron is only the last piece introduced as central atom and co-factor to the heme moiety (114). The same as for porphyrin biosynthesis is applicable for steroid biosynthesis via cholesterol. This biochemical pathway starts from various sources such as glycolysis or beta-oxidation bringing up AcCoA bricks. Assembling these C2 units up to cholesterol is rate-limited by the HMG-CoA-reductase step. Even the arborization to the sexual steroids starting from cholesterol and progesterone involves more than 30 enzymes in the biosynthesis of 17β-estradiol. The whole system is highly regulated and equilibrated (115). A single inborn error in a relevant biosynthetic step may dramatically change this equilibrium. To diagnose an inborn error in the steroid metabolism would need first the identification of genes associated with the disease, commonly assumed by consortia research. The whole genomes of participants with a disease and healthy people need to be sequenced and plotted in a Manhattan plot depicting the abundancy of SNPs (y-axis) against all chromosomes (x-axis). The most abundant SNPs are likely to include the genes responsible for a disease.

**Figure 7:**
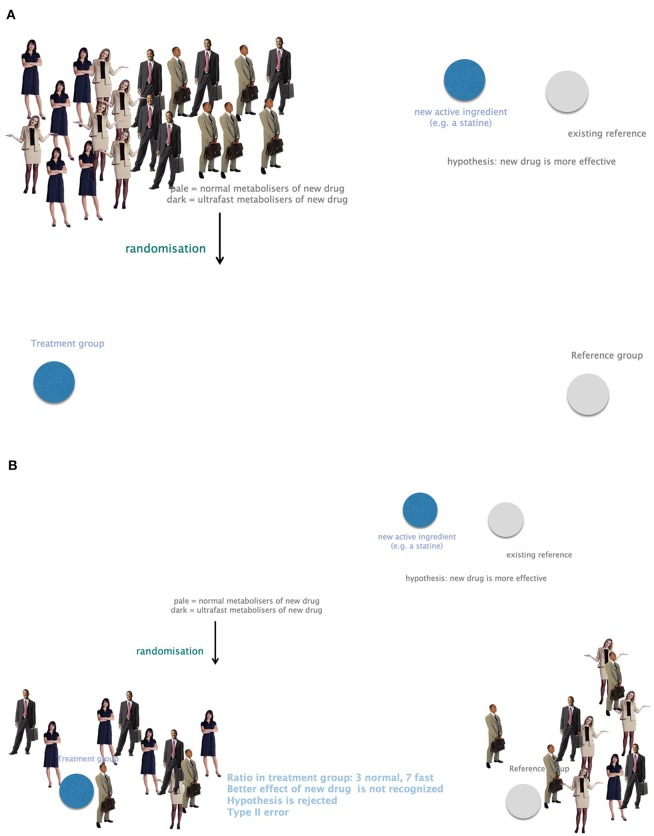
The importance of considering genetic heterogeneity and individual requirements in research and therapy. **(A)** A group of study participants with equally frequent fast and normal metabolizers. **(B)** Small study groups might suffer from unequally distributed fast and normal metabolizers. A presumably more potent new drug could not be recognized in this case. This leads to bias and confounding due to negligence of genetic profiling. Example: the ratio in the treatment group is 3 normal and 7 fast metabolizers. The better effect of the new drug is not recognized. The hypothesis that the new drug is more potent would be rejected. This is a type II error.

Omics technologies include genomics, transcriptomics, proteomics, metabolomics, and many more. Neglecting genes and their impact on phenotypes bears a high risk for biases or confounding for study outcomes. Genetic heterogeneity must therefore be included in clinical trials to omit type II errors arising from unequal randomization to study groups. For example, a new drug presumed to be more potent than a Gold Standard cannot be recognized in a clinical trial in case the treatment group counts more fast metabolizers compared to the control group (Figure 7).

Most of the genomic DNA sequence differences between any two people are common single nucleotide polymorphisms (SNPs) with a minor allele frequency of more than 5% as related to the wild type. The common disease—common variant hypothesis expresses the fact of genetic risk as being due to common SNPs (116). Very rare allele frequency may have a high effect size. They cause Mendeleian diseases of high interest for prenatal medical genetic screening, e.g., phenylketonuria, cystic fibrosis. The penetration of this disease is complete. A mutated gene causes the disease. On the other hand, common allele frequencies with high effects on common diseases are rare. Their penetration is only partial, i.e., an allele is not necessarily determining a disease. Such polymorphisms are neutral or only mildly harmful with respect to survival of a person or of a species as long as alternative metabolic pathways permit bypassing rate-limiting and committed enzymatic reactions. In the band between evolutionary pressure and statistical power are included high effect sizes for rare diseases, low-frequency variants with intermediate effects, and common variants implicated in common diseases. There are many non-communicable diseases in oncology (with some 114 predisposing genes known), psychiatry, obesity, or type 2 diabetes mellitus which can be associated with lifestyle. Rare variants of small effects are difficult to be identified by genetic means, and only rare examples are known for high-effect common variants causing common diseases (117).

Whereas, genome-wide associations can detect reliably genes of Mendeleian inherited disease, it is more complex for syndromes with a polygenic risk. Many of the associated genes with such diseases such as obesity are not causal but only tagging a causal marker. After many generations, tags disappear, the disease-causing alleles persist, a phenomenon known as linkage disequilibrium. Consortia activities contribute most to the increasing knowledge about these diseases. Results cannot simply be transferred to all populations because European ancestry is overrepresented as compared to the global population (118). Another shortcoming of GWAS studies is their statistical character. The higher a sample size, the more significant hits will result. First attempts efforts to associate genes with psychiatric diseases were not successful. In 2009, with 2,601 cases with no hits were detected whereas with 36,000 cases in 2014, 108 hits resulted (119).

As far as ethnicity, the Indian subcontinent shows high incidence cardio-vascular diseases causing four times higher risks of heart attack before the age of 50 and high death rates in 1 of 3 persons fading away before the age of 65. In this population group, high gestational diabetes and metabolic syndrome rates are encountered more often than in any other ethnicity. These diseases are obviously caused by a set of many single nucleotide polymorphisms (SNPs) and thus bear a polygenetic risk arising among others from genes coding for interleukin-6 (IL-6), methylenetetrahydrofolate reductase (MTHFR), intercellular adhesion molecule (ICAM-1), a membrane protein stimulated by retinoic acid (STRA6), or retinol binding protein 4 (RBP4) (120).

## Discussion

### The Relevance of Nutrigenomics and Further OMICS Technologies

As depicted repeatedly above, nutrigenomics becomes relevant for the interpretation of antitumoral effects and further interactions of foodstuff with genes, for potential errors encountered in clinical studies without genomic testings performed on the study population, or in public health as related to non-communicable diseases. Genes code both for metabolism-relevant enzymes and for receptors or targets of medicines and nutrients.

Looking at a person's family roots, it is of interest for both the individual and researcher to know the risks for developing obesity in later life. Obesity is an epidemy worldwide and associated with many health problems, amongst others diabetes, cardiovascular diseases, and cancer. There is a clear (epi)genetic component to a predisposition to obesity. In the last decade, genome-wide studies analyzed hundreds of thousands of SNPs (single nucleotide polymorphisms) and led to a better understanding of the genetic causes of obesity. By 2012, 40 locis were described and associated with obesity and BMI (body mass index). So, slim father and obese mother, or the other way around, whose alleles are found in the siblings? Among at least 65 relevant enzyme's determining energy metabolism, which ones are coding for deficiencies leading to inborn errors associated with obesity albeit in the carbohydrate (glycolysis) or in the lipid metabolism (beta-oxidation) or in in the energy production (citric acid cycle, respiratory chain). The impact of nutrigenomics might be a suitable motivation for nutrition researchers to override some decades of very modest concrete results output and recognize precise relationships between nutritional behavior and disease. Patients suffering worldwide from obesity are waiting for explanations regarding why they are concerned by this disease.

Public health and one-fits-all approaches in nutrition do not satisfy requirements of therapeutic precision in times of personalized nutrition. Physiological and biochemical processes *in vivo* are the result of an individual metabolism which is expressed as encoded by its genes. The individualized capacity of metabolism is affecting qualitative and quantitative uptake of nutrients and xenobiotics in the pharmaco- and nutrient-kinetics steps even before a substrate has found its target. It is continued when the substrate is binding to the fitting physiological receptor. Due to the genetic code this one can be configured qualitatively and quantitatively in a different way than in another person. Nutrigenomics is therefore an emerging research topic to support medical genetics and modern cell and gene therapy of diseases such as spinal atrophy, sickle-cell anemia, many inherited oncological diseases. Whereas, testing a SNP is easy and routine meanwhile, coping with polygenic risks will remain a challenge for the next decades.

What has been widely practiced in pharmacotherapy is urgently needed to be applied in nutritional sciences. Traditional nutrition research did not succeed to put out breakthroughs in the past decades. Epidemiological and qualitative questionnaires-dominated methodologies suffered from serious biases and confounding. Generalizations to whole populations were made although results were just applicable for a defined study population and regional behaviors.

### Summary of Main Findings

Public health dominated research is more and more competed by personalized research approaches. The mystery of biochemical pathways applies commonalities as long as the mechanisms and principles of anabolism and catabolism are in the focus. On a quantitative level however, a person's metabolic capacity depends on his or her individual genetic pattern.

Absorption of nutrients and xenobiotics is mainly an individualized procedure depending on a human being's metabolic capacity. This in turn varies with age, gender, ethnicity, and inherited metabolic performance. It is gender-, age-, and ethnic-dependent whether an individuum can be attributed to a certain group of metabolizers. Depending on the age and gender, the ratios of body compartments vary and therefore the distribution according to the hydrophilic—lipophilic profile of a substrate. Metabolism is discussed mainly in terms of the hepatic part due to its predominance, although it happens also in extra hepatic hormone-dependent tissues. The role of CYP450 isoenzymes is a central and determining one as it does not only act as monooxygenase to detoxify but also to biosynthesize from metabolites further hormonal active substances such as estrogens. The mechanisms of action and the catalytic cycles themselves depend on further enzymes, which are themselves highly regulated. Excretion is often discussed as an isolated procedure. However, the Bateman function describing the course of plasma level is in fact the sum of absorption, distribution, metabolism, and excretion.

On a pharmaco- and nutrient-dynamic level, aromatase as central interest of this review and target of the precursors of estrogens is also a main target of medicines used in breast cancer treatment. Nutrients of the flavonoid group are partially structurally similar to estradiol precursors testosterone and androstendione. They have a relevant potential to prevent breast cancer and to be used as adjuvants in pharmacotherapy.

Knowing biochemistry of pharmacokinetics (LADME) and pharmacodynamics as well as biosynthesis of the main carriers and enzymes of these procedures is not enough. As the metabolic capacity of a person is depending on the genome and various circumstances relevant for the expression of these genes, non-communicable diseases cannot be prevented in the population by treating specific pathways without considering genetics and epigenetics.

Genetic regulation of metabolism is a complex procedure affecting multiple pathways. All ATP-dependent metabolic steps need an effective glycolysis (for pyruvate and acetyl-CoA production), citric acid cycle (for electron harvesting), and respiratory chain/oxidative phosphorylation (for ATP replenishment). Fine-tuning this kind of energy production depends therefore on co-enzymes (B-vitamins) and co-factors (mainly iron, copper). Energy production in combination to gene regulation by “anti-oxidant” nutrients are key elements for cancer prevention and add-on therapies parallel to conventional cytotoxic protocols.

In most of the cases, to obtain an entirely well-resolved picture of a pathogenic situation, there is a need to know underlying biochemical pathways. Therefore, an inborn error of metabolism ending up in manifest breast tumor needs to be assessed within the affected tissue, the involved physiological substrates and metabolites, the capacity of enzymes involved in these steps, the capacity of underlying biochemical pathways, and finally the genotype and phenotype of the patient. It may even be wise to evaluate environmental circumstances, epigenetics, and immunological enhancers or inhibitors.

With the infinite number of recombination of the genes, each person is unique, and all diagnosis must consider the genetic patterns with numbers of SNPs leading to differences in liberation, absorption, distribution, metabolism, and excretion of nutrients from a food matrix. A variable uptake of bioactives is the first quantitative difference in nutritional therapy. Receptors of any two persons may differ in their number and in their structure. These differences potentiate the quantitative differences of a response to nutrients intake.

### Limitations

Although scientific publications have been carefully selected, not all are arising from evidence-based findings of clinical trials. Some interpretations and statements are based on biochemically and pharmaceutically accepted theories and state of the art. Commonalities and principles have been proved over centuries. Evidence can only be generated if methodologies are convincing. However, many publications are limited to regional or ethnical frames and cannot be transferred to other ethnicities. And many studies are erroneous, because genetic profiling has been neglected. It is preferable to interpret and reevaluate carefully some findings from early publications as soon as new knowledge is emerging on a novel research field. Often, early findings remain standards although new insight hints at pitfalls in methodologies.

The statements made in this article might be countered in later research activities.

## Conclusion

Nutrition science needs to consider the genome of an individual to truly find answers to nutrition-derived non-communicable diseases. All steps from intake to effect are genetically encoded. Genetic tests to detect risks for pathologies and determine optimal individual diets should be considered in nutrition research and practices. With current GWAS approaches, inherited errors of metabolism are identified and ideally treated effectively as long as the one variant—one disease hypothesis is applicable. It is much more difficult to get a precise genetic profile for multifactorially caused non-communicable diseases. Polygenic risks are feasible but diagnostic tools are not yet available in a desired extent. As to whether explanations beyond pure chemical mechanisms of action are described in the literature, the answer is that neither authors, nor providers of genetic testing kits are going into the details needed for a truly personalized nutritional medicine. The next step with sequencing the whole exome is on the threshold of becoming routine diagnosis. The further step of sequencing the entire genome will bring the desired details such as regulation option by intron-coded miRNA and further nucleic acid or protein fragments. On this way, actual genetic testing is only an intermediate sojourn on the way to whole genome sequencing.

## Data Availability Statement

The raw data supporting the conclusions of this article will be made available by the authors, without undue reservation, to any qualified researcher.

## Author Contributions

HJ and LS-R contributed equally to the general nutritional medical contents and nutrigenomics. HJ lead the paragraphs oriented to clinical nutritional therapy, biochemistry, and mechanisms of action. LS-R lead the paragraphs on biology and public health nutrition.

### Conflict of Interest

The authors declare that the research was conducted in the absence of any commercial or financial relationships that could be construed as a potential conflict of interest.

## Footnotes

^1^COST Action FA1403 (POSITIVe, https://www6.inra.fr/cost-positive).
